# Zoledronic acid inhibits pulmonary metastasis dissemination in a preclinical model of Ewing’s sarcoma via inhibition of cell migration

**DOI:** 10.1186/1471-2407-14-169

**Published:** 2014-03-10

**Authors:** Guillaume Odri, Pui-Pui Kim, François Lamoureux, Céline Charrier, Séverine Battaglia, Jérôme Amiaud, Dominique Heymann, François Gouin, Françoise Redini

**Affiliations:** 1 INSERM, Equipe Ligue Contre le Cancer 2012, UMR-957, Nantes F-44035, France; 2 Faculté de Médecine, Laboratoire de physiopathologie de la résorption osseuse et thérapie des tumeurs osseuses primitives, Université de Nantes, EA3822, Nantes F-44035, France; 3 Service d’orthopédie, CHU Hôtel Dieu, Nantes F-44035, France; 4 INSERM UMR957, Faculté de Médecine, 1 rue Gaston Veil, 44 035, Nantes Cedex 1, France

**Keywords:** Ewing’s sarcoma, Zoledronic acid, Lung metastases, Animal models

## Abstract

**Background:**

Ewing’s sarcoma (ES) is the second most frequent primitive malignant bone tumor in adolescents with a very poor prognosis for high risk patients, mainly when lung metastases are detected (overall survival <15% at 5 years). Zoledronic acid (ZA) is a potent inhibitor of bone resorption which induces osteoclast apoptosis. Our previous studies showed a strong therapeutic potential of ZA as it inhibits ES cell growth in vitro and ES primary tumor growth in vivo in a mouse model developed in bone site. However, no data are available on lung metastasis. Therefore, the aim of this study was to determine the effect of ZA on ES cell invasion and metastatic properties.

**Methods:**

Invasion assays were performed in vitro in Boyden’s chambers covered with Matrigel. Matrix Metalloproteinase (MMP) activity was analyzed by zymography in ES cell culture supernatant. In vivo*,* a relevant model of spontaneous lung metastases which disseminate from primary ES tumor was induced by the orthotopic injection of 10^6^ human ES cells in the tibia medullar cavity of nude mice. The effect of ZA (50 μg/kg, 3x/week) was studied over a 4-week period. Lung metastases were observed macroscopically at autopsy and analysed by histology.

**Results:**

ZA induced a strong inhibition of ES cell invasion, probably due to down regulation of MMP-2 and −9 activities as analyzed by zymography. In vivo, ZA inhibits the dissemination of spontaneous lung metastases from a primary ES tumor but had no effect on the growth of established lung metastases.

**Conclusion:**

These results suggest that ZA could be used early in the treatment of ES to inhibit bone tumor growth but also to prevent the early metastatic events to the lungs.

## Background

Ewing sarcoma (ES) is the second most frequent primary bone malignancy in adolescents and young adults with a reported annual incidence rate of 2.93 cases/10^6^ in the interval from 1973 to 2004 [1]. ES is defined by a chromosomal translocation involving the EWS gene on chromosome 22 with a gene of the ETS family located on different chromosomes [2], leading in 85% of cases to the EWS-FLI1 translocation t(11;22)(q24;q12), whereas the EWS-ERG gene occurs in the majority of the remaining 15% of EFTs. Detection of the translocations allows a specific molecular diagnosis. Despite the impressive improvement of survival in the last decades using multimodal approaches, 5-year overall survival (OS) of ES patients with localized disease remains at 70% [3], dropping down to <15% in patients with multifocal primary disease or with early relapse [4]. At diagnosis, metastases are detected in 15% to 33% of patients [5,6], with survival rates from 9% to 41% [7,8]. Patients with primary pulmonary metastases fare better than patients with primary bone and/or bone marrow (BM) involvement [9]. In the absence of chemotherapy, approximately 90% of patients die from disease following definitive surgery, suggesting that the vast majority of patients have micro-metastatic disease at presentation [10,11]. Therefore, new therapies have to be developed to inhibit metastasis dissemination in ES.

Bisphosphonates (BPs) are pyrophosphate derived molecules which selectively concentrate at the bone resorption surface [12], induce osteoclast apoptosis resulting in inhibition of bone resorption [13]. In addition, they inhibit adhesion, invasion and proliferation, and induce apoptosis in a variety of human tumor cell lines in vitro such as breast, myeloma, pancreas, melanoma, prostate cancer and osteosarcoma [14]. Among all BPs tested, Zoledronic Acid (ZA), one of the third generation nitrogen containing BPs, shows the greatest inhibitory effects on both osteoclast activity and tumor cell proliferation. Used since several years for the treatment and prevention of osteoporosis, its application is now extended to reduce skeletal morbidity in patients with malignant bone disorders [15]. It is increasingly used alongside anticancer treatments to prevent skeletal complications and relieve bone pain. Concerning primary bone tumors, ZA has been associated to conventional chemotherapy and surgery in the French OS2006 phase III clinical trial for osteosarcoma treatment, after promising preclinical results had been found on survival and tumor growth [14,16]. ZA has also been found to inhibit bone and visceral metastases development in several types of cancer [17].

In Ewing’s sarcoma, ZA has been shown to inhibit proliferation on ES cell lines in vitro and to slow the tumor growth in a mouse ES model in bone [18]. Because ES patients harbor micrometastases very early in the disease, we thought that ZA could have an effect to cure or prevent these metastases. The aim of this study was to determine the effect of ZA on ES cell migration and metastatic properties in vitro through migration and invasion assays and gelatin zymography, and in vivo in a mouse ES model of spontaneous pulmonary metastases.

## Methods

### Cell lines and culture

The human Ewing’s sarcoma A-673 cell line was provided by Dr S. Burchill (Children Hospital, Leeds, UK) and the TC-71 cell line by Dr. O. Delattre (INSERM U830, Institut Curie, Paris, France). These two cell lines were chosen as they respond differentially to ZA: A-673 is sensitive (IC50 = 3 μM) and TC-71 more resistant (IC50 = 100 μM). A-673 and TC-71 cell lines were cultured respectively in DMEM (Dulbecco’s Modified Eagle Medium, Biowhittaker) and RPMI (Roswell Park Memorial Institute, Biowhittaker) medium both with 10% fetal bovine serum (FBS, Hyclone, France). All cultures were performed under laminar flow hood (PSM Securiplus, Astec France) in controlled mycoplasma free environment. The cells, initially seeded at the concentration of 10^4^cells/mm^2^, were incubated at 37°C with humidity saturated controlled atmosphere and 5% CO_2_. At confluence, cells were detached with trypsine-EDTA [Biowhittaker, Trypsine: 0.5 g/L; EDTA (Ethylene Diamine Tetraacetic Acid): 0.2 g/L]. Trypsine was neutralized by adding FBS-containing medium and cells were collected after centrifugation at 1600 rpm.

### Invasion assay

A-673 Ewing’s sarcoma cells were treated by 20 μmol/L ZA [1-hydroxy-2-(1H-imidazole-1-yl) ethylidene-bisphosphonic acid supplied as the disodium salt by Novartis Pharma AG] during 24 h. Invasion of cultured cells was analyzed using Boyden’s chambers (8 μm pores, Becton Dickinson Labware) covered by polyethylene terephtalate membrane with Matrigel® coating (2 μg/100 μL/well in cold PBS) in 24-wells plate (Multiwell™ 24, FALCON®). At the end of the 24 h-period, viable cells were counted (by trypan blue exclusion) and the same number of ZA treated and non treated viable cells (4.10^4^) were seeded in the upper compartment of 500 μL cups in 1% FBS medium. The chamber was immerged in 700 μL of 10% FBS medium and left 48 h for incubation at 37°C in 5% CO_2_ humidified atmosphere. Non invasive cells were removed and invading cells present on the inferior surface of the membrane were fixed by 3% PFA (ParaFormAldehyde) and stained by methylene blue. After drying, the invasive cells were counted with 10× microscope in 5 microscopic fields using DP controller, DP manager software (Olympus). All experiments were repeated 3 times in duplicates and invasion is expressed by mean number of cells / field.

### Gelatin zymography

Metalloproteinase activity was analyzed by gelatin zymography. A-673 and TC-71 cells were cultured and treated for the last 24 h of culture (at subconfluent state) with increasing concentrations of ZA (5 to 100 μM) in serum free medium. This treatment period did not alter final cell number as determined by trypan blue exclusion (not shown). Then same supernatant volume was electrophoresed in 10% SDS polyacrylamide gels containing 1 mg/ml gelatin. Gels were then incubated in 2.5% Triton X-100 to remove SDS, washed briefly in distilled water and then incubated in 50 mM Tris–HCl, 10 mM CaCl_2_ (pH 7.5) overnight at 37°C. The gels were then stained with 0.25% (w/v) Coomassie brilliant blue and destained with 10% isopropanol in 10% acetic acid. The gelatinolytic activity was identified as transparent bands in the Coomassie brilliant blue–stained background.

### In vivo experiments

#### Animal ethics

All procedures involving animals were conducted in accordance with the Directive 2010/63/EU of the European Parliament and the Council of the 22/09/2010 on the protection of animals used for scientific purposes. The protocols presented in this study were approved by the French ethics committee (CEEA PdL. 06) with the protocol number 2010.34.

#### Mouse models of ES

To study the effect of ZA on the metastatic ability of ES, an intra-osseous (IO) model was developed as described previously [18]. Four-week-old female athymic mice were purchased from Janvier breeding (St Genest, France). Mice were anesthetized by inhalation of a combination isoflurane/air (1.5%, 1 L/min) and they received buprenorphine after the tumor cell injection (0.05 mg/kg; Temgesic®, Schering-Plough). Mice were randomly assigned to treatment groups 1 day after the tumor cell injection. The tumor volume was calculated by using the formula L × (l^2^)/2, where L and l represent respectively the longest and the smallest perpendicular diameter.

#### Lung metastases experiments

First experiment: 30 mice were injected with 1 million A-673 cells in the right tibia (intra-osseous injection: IO) at day 1 and were amputated at day 2. Amputation was realized under anesthesia, by disarticulation of the hip joint. The mice received 0.05 mg/kg buprenorphine all along the experiment. They stayed individually until day 45, when they were sacrificed and lungs were collected for histological analysis.

Second set of experiments (24 mice): After IO injection of 1 million A-673 or TC-71 cells in the right tibia, half of mice were randomly assigned to the control group (n = 12), which received subcutaneous PBS injections (3×/week), or to the treated group (n = 12) which received subcutaneous ZA 50 μg/kg (3×/week) starting day 2 after tumor cell inoculation. Mice were euthanized when tumor volume exceeded 2500 mm^3^ or when mice showed signs of lung metastases development (respiratory distress, weakness, weight loss, dorsal kyphosis). Lungs were collected for histological and macroscopic analysis: the lungs were categorized according to the size (big or small) of the metastases. Mice were excluded from analysis when no tumor developed in the tibia or when the IO injection failed or was performed intra-muscularly.

Third experiment (30 mice): Mice were injected with A-673 ES cells at day 1 and 3 were sacrificed at early time points before primary tumor could be detected (day 3, 7, 10, 13 and 17 after tumor cell injection). A group of 15 mice was treated by subcutaneous injection of ZA 50 μg/kg 3×/week starting at day 2. A control group of 15 mice received subcutaneous PBS injections. Three mice were euthanized in each group at each endpoint and lungs were collected for histological analysis.

#### Immuno-histochemical analysis

Immuno-staining with anti-human CD99 antibody was performed on collected lungs from ZA treated and non treated mice. All samples were included in paraffin and 2–4 μm cuts were performed with a microtome (Leica RM 2255, Leica microsystème SAS, France). The samples were automatically deparaffined (HMS740 automatic: 3 × 5 mn OTTIX PLUS, 3 × 5 mn Ethanol 100°, 1 × 5 mn Ethanol 95°, 1 × 5 min Ethanol 80°, 3 × 5 mn in distilled water), and rinsed in TBS 1× pH = 7.6 Tween 0.05% at room temperature. Endogeneous peroxydases were blocked by H_2_O_2_ 3% 15 min at room temperature and nonspecific sites were blocked by Goat serum 5%, BSA1% diluted in TBS 1× pH = 7.6 Tween 0.05%. Samples were incubated with the primary mouse anti CD99 antibody (diluted at 1:50) at room temperature and rinsed in TBS 1× pH = 7.6 Tween 0.05%. Secondary biotinylated goat anti mouse antibody (Dako, E0433) diluted at 1:200 was applied 30 min at 37°C and rinsed in TBS 1× pH = 7.6 Tween 0.05%. The samples were then incubated in streptavidine/ peroxydase (Dako, P0397) diluted at 1:200 in TBS. The substrate was applied 1–10 min in obscurity and rinsed. The samples were counterstained in HMS740 with hematoxyllin Gill. Slides were then mounted and ready for observation.

#### Statistical analysis

All in vitro experiment were realized 3 times. Numbers of cells per field mean counts were compared by a non parametrical Wilcoxon test. In vivo, mean tumor volumes were compared using Kruskal-Wallis test. The size of lung metastases categorical variable was analyzed by Fisher’s exact test. The difference was considered significant at p < 0.05.

## Results

### Zoledronic acid inhibits Ewing’s sarcoma cell invasion in vitro

**Figure 1:**
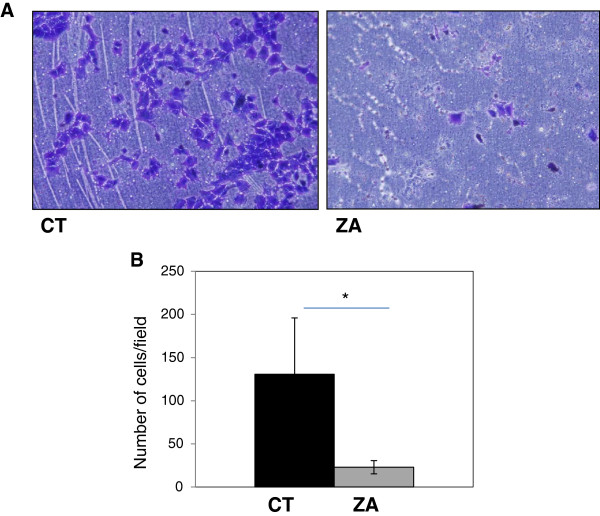
**Effect of zoledronic acid (ZA) on Ewing’s sarcoma cell invasion through Matrigel.** Human A-673 ES cells were seeded and cultured for 24 hours in the presence of ZA. Then 4.10^4^ cells were placed on the top of the Boyden’s chamber and left to invade the Matrigel 48 h without ZA. **A**. Microscopic observation (×10) of ES cells on the bottom of the Boyden’s chamber at the end of the 24 h. **B**. mean number of cells /field after invasion assay in Boyden’s chamber recovered by Matrigel. (*: p < 0.05).

To determine whether ZA could affect tumor cell invasion, assays were realized in Boyden’s chambers covered with Matrigel. Because ZA affects tumor cell proliferation, only the surviving cells (determined by trypan blue exclusion test) after 24 h ZA 20 μmol/L treatment were used and compared to non treated cells. The same number of treated and non treated viable cells (4.10^4^) was thus seeded in the top of Boyden’s chambers and left for 24 hours. On the bottom side of the membrane an average of 130.75 cells/ field was counted for the non treated cells versus 22.9 cells/ field for the ZA treated cells (Figure 1A and B), the difference being statistically significant (p <0.001). Therefore, ZA 20 μmol/L significantly inhibits A-673 ES cell line invasion through a Matrigel membrane in vitro.

### Zoledronic acid inhibits matrix metalloproteinase (MMP) 2 and 9 activity

**Figure 2:**
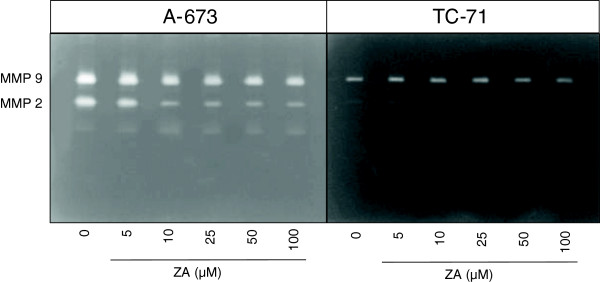
**Effect of zoledronic acid (ZA) on MMP activity analyzed by gelatin zymography.** MMP-2 and MMP-9 activities were evidenced at the corresponding molecular weight. Subconfluent A-673 and TC-71 human ES cell lines were cultured in the presence or absence of zoledronic acid at increasing concentrations (5 to 100 μM) for the last 24 h of culture. The supernatant was then harvested and the same volume submitted to electrophoresis in a gel containing gelatin (see Methods section).

In order to determine whether ZA-induced inhibition of ES invasion was due to impaired MMP-2 and −9 activities, zymography assay was performed on culture supernatants of Ewing’s sarcoma cells treated or not with ZA. At subconfluent state, TC-71 and A-673 cells were cultured for the last 24 h with or without ZA in serum free conditions. At the end of this period, cell cultures were confluent, and no differences in cell number could be determined. Same volume of culture medium was collected and analyzed by gelatin zymography. Zymographs presented in Figure 2 showed that ZA inhibits MMP9 and MMP2 activity in both ES cell lines studied. This inhibition is ZA dose-dependent especially for the A-673 cell line, and is more significant when considering MMP-2 than MMP-9 activity.

These interesting results prompted us to complete this study by an in vivo approach, in order to determine whether ZA could inhibit pulmonary metastasis dissemination in relevant ES models of metastases. Indeed, we previously demonstrated that ZA was able to inhibit Ewing’s sarcoma tumor progression in bone, and the present encouraging results on Ewing’s sarcoma cell migration suggest that ZA could also influence metastasis formation in vivo.

### Development of an ES model of pulmonary metastasis dissemination

To develop such in vivo studies, a relevant model of spontaneous lung metastases was needed. From previous work showing that pulmonary metastases could only be observed in models induced by intra-tibia tumor cell injection, this model was chosen in the present study (no pulmonary metastases could be observed when the ES cells were injected in soft tissue). However, because very early lung metastases could be observed in this intra-osseous model, we hypothesized they could result from direct tumor cell emboli during the injection. Indeed, 0 to 50% of the mice died of pulmonary distress within minutes after the injection, possibly due to massive pulmonary embolism. To demonstrate this hypothesis, a first experiment was realized in which mice were amputated at day 1 after intraosseous injection of 1.10^6^ A-673 ES cells in the medullar cavity of the tibia and left to live until day 45, at which time they were sacrificed. In this experiment, none of the mice died immediately after the injection. Three mice out of 30 developed lung metastases, and all of these were “big” (one diameter > 5 mm determined by histology; data not shown). No small metastases were found. Thus, we must be aware that intraosseous injection alone can induce experimental metastases, determined as “big” metastases at sacrifice (i.e. when primitive tumor volume reaches 2500 mm^3^, approx. 45–50 days after tumor cell injection) that are not representative of spontaneous metastases disseminating from an established primary tumor.

### Effect of zoledronic acid on spontaneous lung metastasis dissemination

**Figure 3:**
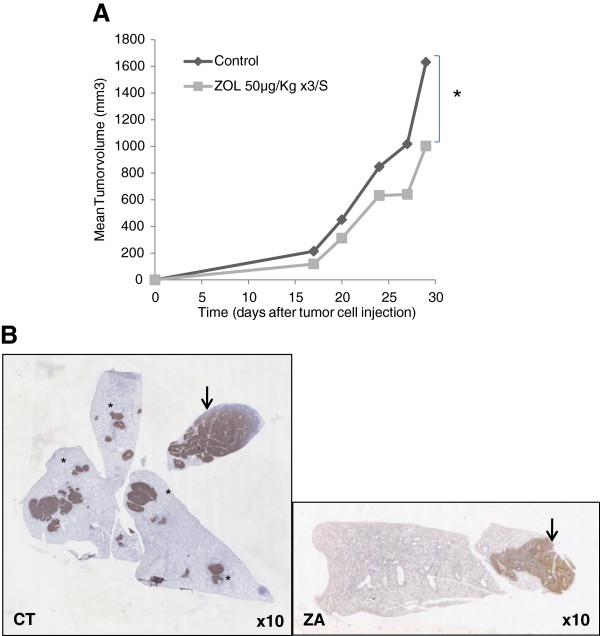
**Effect of ZA on tumor progression and metastasis dissemination. A**. Comparison of tumor volume evolution between ZA (50 μg/kg, 3 times a week during 4 weeks) treated and non treated (PBS) nude mice in the A-673 Ewing’s sarcoma model; **B**: Histological comparison of lung metastases in mice 30 days after A-673 ES tumor cell injection in the medullar cavity of the tibia. ES cells were revealed by positive CD99 immunostaining. (left: Control mice, magnitude × 10; right: ZA treated mice, magnitude × 10). Both small (spontaneous, stars) and big (experimental, arrow) metastases can be seen in control mice whereas only a big one (arrow) is seen in ZA treated mice. These histology analyses are representative data of at least 6 mice/group.

The dose and time schedule used in the present study reproduce those used in the OS2006 clinical trial for pediatric patients (50 μg/kg, every 4 weeks). We first confirmed that this protocol also induces a significant inhibition of primary Ewing’s sarcoma progression in bone at day 29 (p < 0.05, Figure 3A) as it has been published with the previous protocol used (100 μg/kg, twice a week, [18]). To determine whether ZA could affect spontaneous pulmonary metastasis formation, mice received intra-tibia injection of 1.10^6^ A-673 or TC-71 ES cells and divided into 2 groups (n = 12/group): the control group treated with PBS and the other treated with ZA 50 μg/kg 3 times a week. Mice were sacrificed when the primary tumor reached 2500 mm^3^ (around day 45–50 after tumor cell injection) and the lungs were collected for histology analysis. Lungs from control mice exhibit both “big” (defined nodules with one diameter > 5 mm) and “small” (defined as < 5 mm) metastases within the same lungs (Figure 3B, left: arrows: big metastases and stars: small metastases), big metastases reflecting “false” experimental metastases due to the initial tumor cell injection, and the smaller ones corresponding to newer spontaneous metastases disseminating from the primary tumor. ZA-treated mice showed also big metastases but very few or no small ones (Figure 3B, right). The macroscopic analysis results of the different experiments are summarized in Table 1a (A-673 Ewing’s sarcoma model) and 1b (TC-71 Ewing’s sarcoma model): ZA treated mice had significantly less small metastases than the untreated ones (p < 0.05), although no significant change was observed in the amount of large metastases. These results suggest that ZA limits metastases arising spontaneously from the primary bone tumor (small metastases).

**Table 1:** Comparison of the number and percentage of mice in each group according to the size of the metastases found in the A-673 (a) and the TC-71 (b) models

**a**			
		**N**	**%**
Total number of mice		24	
Dead mice at injection		3	12.5
Randomized mice		21	
Control group	Total number of mice in group	10	
	Intra-muscular injection	0	0
	Mice with no metastasis	1	10
	Mice with big metastasis	4	40
	Mice with small metastasis	8	80
	Mice with big and small metastasis	3	30
ZA treated group	Total number of mice in group	11	
	Intra-muscular injection	1	9
	Mice with no metastasis	2	18
	Mice with big metastasis	8	73
	Mice with small metastasis	2	18
	Mice with big and small metastasis	2	18
**b**			
		**N**	**%**
Total number of mice		24	
Dead mice at injection		5	21
Randomized mice		19	
Control group	Total number of mice in group	9	
	Intra-muscular injection	0	0
	Mice with no metastasis	2	22
	Mice with big metastasis	3	33
	Mice with small metastasis	7	78
	Mice with big and small metastasis	3	33
ZA treated group	Total number of mice in group	10	
	Intra-muscular injection	1	10
	Mice excluded because no tumor	1	10
	Mice with no metastasis	4	40
	Mice with big metastasis	2	20
	Mice with small metastasis	2	20
	Mice with big and small metastasis	0	0

### Effect of Zoledronic acid on experimental lung metastases

**Figure 4:**
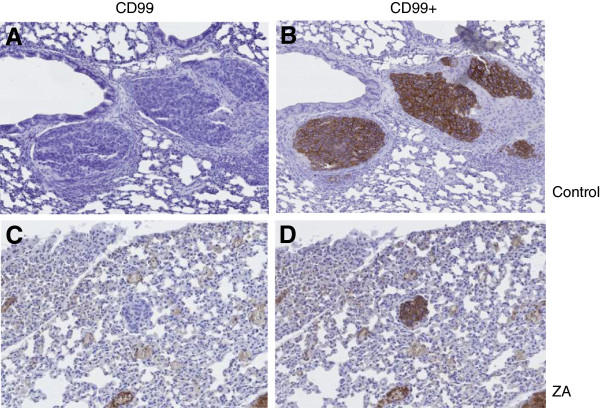
**Histological observation of early metastases after intra-tibial injection of A-673 ES cells. A-B**: control mice treated with PBS: observation 7 days after tumor cell injection (A: CD99-, B: CD99+). **C-D**: ZA treated group: observation 7 days after tumor cell injection (C: CD99-, B: CD99+). The metastatic ES cells appear in brown on the CD99+ staining panels **(B and D)** (all magnitude: × 130).

We also studied the effects of ZA on early development of experimental lung metastases. Thirty mice received injection of 1.10^6^ A-673 ES cells in the medullar cavity of the tibia, 15 being treated with ZA 50 μg/kg three times a week and the others with PBS (controls). Three mice were euthanized at different endpoints early before tumor detection at the primary site (3, 7, 10, 13 and 17 days after tumor cell injection), to determine whether ZA could act on early metastasis development, corresponding to direct dissemination in the blood circulation after injection (“false” experimental metastases). Metastases could be observed in this intra-osseous injection model in both groups, with no significant difference in the size, the number and the delay of appearance, suggesting that ZA has no effect on early experimental lung metastases development (Figure 4).

## Discussion and conclusions

The objective of this study was to characterize the effect of zoledronic acid on Ewing’s sarcoma cell invasion and pulmonary metastasis dissemination by both in vitro and in vivo approaches.

An inhibitory effect of ZA was observed on ES cell invasion in vitro, as well as an inhibition of MMP-2 and −9 activities. ZA has been previously reported to decrease cell migration and invasion for tumor cells in prostate cancer, pancreatic carcinoma [19], osteosarcoma [20], and breast cancer cells [21], but also for non neoplasic cell types such as osteoclasts, osteoblasts, fibroblasts [22], endothelial progenitor cells, oral epithelial cells, or vascular smooth muscle cells [23]. Because MMP-2 and −9 are considered as the proteinases mostly involved in the invasion process, several studies provide evidence for an effect of BPs in preventing tumor cell invasion through MMP decreased expression and activity, but also by a direct inhibitory effect on MMP proteolytic activity through zinc chelation [21]. In our experiments, we carefully distinguished between ZA effect on ES cell proliferation and on cell invasion. For that purpose, ES cells were treated during 24 hours with ZA, and only the same number of surviving cells was used in the invasion assay. In addition, ZA being not present during the invasion assay, the observed inhibition of cell invasion could not be due to ZA-induced cell death or to the inhibition of MMP activity through zinc chelation.

The second part of our work focused on the potential in vivo effect of ZA on pulmonary metastasis formation. We first needed to develop a relevant model of spontaneous pulmonary metastases. Only one study reported an experimental model of bone metastases in Ewing’s sarcoma induced by intra-veinous injection of TC-71 cells, in which lung metastases were also observed [24]. In our laboratory, two main approaches have been used to develop primary ES tumors in mice: tumor cell injection in soft tissue or inside the medullar cavity [18]. From these previous experiments, no metastasis formation could be observed in the soft tissue model whereas many lung metastases were detected in the intra-osseous induced model. However, intra-tibial injection of ES cells in nude mice was shown to induce early experimental metastases in 10% of cases, likely as if the cells were injected directly into the bloodstream. In some cases, mice immediately died from respiratory distress, probably due to a massive tumor cell embolism into the pulmonary veins. This is in accordance with the presence of very early lung metastases found in the mice as early as day 3 after tumor cell injection in the present study. It is also in accordance with the presence of lung metastases in mice which were amputated at day 1, which make it impossible that these metastases arise from disseminating metastatic cells from a well implanted primary tumor. Thus, during intra-tibial injection of the ES cells, cell embolisms may directly go to the lungs, creating “false” experimental metastases. This has to be taken into account while studying lung metastases using this intra-osseous model.

Two ES models were compared in this study: one induced by a sensitive (A-673) and one by a resistant (TC-71) cell line. The aim was to determine whether the ZA effects on invasion, migration and metastasis formation was dependent or not on its effect on tumor cell proliferation. Results showed that in both models, ZA diminished the formation of “small” spontaneous metastases, suggesting that ZA may exert direct effect on invasion independent of tumor cell proliferation.

Two recent studies reported that ZA could increase lung metastases of osteosarcoma after intra-tibial injection in nude mice, but none of those studies explain the difference between experimental and spontaneous metastases that is a real limitation of this approach [25,26]. In the present study, ZA prevents spontaneous lung metastases spreading from the initial tumor as shown in the second set of experiments, but had no effect on the “false” experimental metastases. Histological analysis found very few to no small metastases in lungs from ZA treated mice. These small metastases, because not present in the amputation model, arise later during the tumor development and represent spontaneous metastases, needing cell migration and invasion to develop. Several studies in mouse models have shown that BPs treatment can inhibit bone metastasis development in vivo. Indeed, BPs are now commonly given to prevent bone metastases in many epithelial cancer types (breast, prostate). Preclinical studies reported that BPs may prevent visceral metastases of breast cancer [27] and lung metastases of osteosarcoma [16]. However, no studies have reported a direct effect of ZA on established lung or other visceral metastases. This is probably due to the fact that serum concentrations of ZA are very low, as ZA immediately binds to the bone matrix after injection. Therefore, we can hypothesized that ZA may prevent the metastatic process by itself, but has no effect on the metastases themselves when they are established.

Overt metastases are associated with a poor prognosis in Ewing’s sarcoma, but patients without overt metastases frequently harbor micrometastatic disease at presentation. Circulating tumor cells can often be identified in patients using RT-PCR or flow-cytometry techniques. This suggests that the metastatic potential of Ewing’s sarcoma exists at an early stage during tumor development [11] and that a very early event such as the initiating oncogenic event might influence cell proliferation and capacity for metastasis. Ewing’s sarcomas are characterized by a specific translocation between the EWS gene and a gene from the ETS family. It appears that the loss of cell adhesion needed to promote tumor cell dissemination might be induced by the EWS/FLI1 oncogene itself rather than via an accumulation of stepwise mutations [28]. Furthermore, cell migration is similarly inhibited by EWS/FLI expression, suggesting that dissemination occurs via a “passive” rather than via an active process that can be observed in epithelial tumors undergoing epithelial to mesenchymal transition [28]. Several studies have also reported that ES cells exhibit an anoikis resistant phenotype [29]. Therefore, it is probably through their exposure to ZA in bone that ES cells invasion potential is altered, even though they continue to disseminate through the loss of adhesion, resulting in a reduction of spontaneous lung metastases.

In conclusion, associated with previous results showing that ZA is able to inhibit primary tumor growth in a bone model of ES, the present results strengthen the therapeutic interest of ZA in Ewing’s sarcoma, as ZA could be associated early to chemotherapy for ES patients to prevent ongoing spontaneous metastases dissemination from the existing primary bone tumor.

## Abbreviations

BP: Bisphosphonate; ZA: Zoledronic acid; ES: Ewing’s sarcoma; MMP: Matrix metalloproteinase.

## Competing interests

The authors declare that they have no financial and non-financial competing interests.

## Authors’ contributions

GAO and P-PK carried out the in vivo studies, the migration and zymography studies, FL participated to the in vivo studies, CC and JA participated to the histology studies, SB participated to the in vivo studies. FG and DH have been involved in revising critically the manuscript for important intellectual content. FR had made substantial contribution to the study conception and design, had been involved in drafting the manuscript. All authors read and approved the final manuscript.

## Pre-publication history

The pre-publication history for this paper can be accessed here:

http://www.biomedcentral.com/1471-2407/14/169/prepub
